# AutoGPA: An Automated 3D-QSAR Method Based on Pharmacophore Alignment and Grid Potential Analysis

**DOI:** 10.1155/2012/498931

**Published:** 2012-11-26

**Authors:** Naoyuki Asakawa, Seiichi Kobayashi, Junichi Goto, Noriaki Hirayama

**Affiliations:** ^1^Science and Technology Systems Division, Computational Science Department, Ryoka Systems Inc., 1-28-38 Shinkawa, Chuo-ku, Tokyo 104-0033, Japan; ^2^Basic Medical Science and Molecular Medicine, Tokai University School of Medicine, 143 Shimokasuya, Isehara, Kanagawa 259-1143, Japan

## Abstract

3D-QSAR approach has been widely applied and proven to be useful in the case where no reliable crystal structure of the complex between a biologically active molecule and the receptor is available. At the same time, however, it also has highlighted the sensitivity of this approach. The main requirement of the traditional 3D-QSAR method is that molecules should be correctly overlaid in what is assumed to be the bioactive conformation. Identifying an active conformation of a flexible molecule is technically difficult. It has been a bottleneck in the application of the 3D-QSAR method. We have developed a 3D-QSAR software named AutoGPA especially based on an automatic pharmacophore alignment method in order to overcome this problem which has discouraged general medicinal chemists from applying the 3D-QSAR methods to their “real-world” problems. Applications of AutoGPA to three inhibitor-receptor systems have demonstrated that without any prior information about the three-dimensional structure of the bioactive conformations AutoGPA can automatically generate reliable 3D-QSAR models. In this paper, the concept of AutoGPA and the application results will be described.

## 1. Introduction

There are two major types of *in silico* drug discovery techniques: structure-based and ligand-based techniques. Quantitative structure-activity relationship (QSAR) approach only based on biological activities and chemical structures of a series of molecules with the modest biological activities is one of the ligand-based techniques. The QSAR approach explicitly considering three-dimensional shape of molecules is called 3D-QSAR. The CoMFA method proposed by Cramer et al. [1] is one of the 3D-QSAR approaches which has been widely applied and proven that the 3D-QSAR approach is better than the traditional QSAR one. The CoMFA method is based on the idea that biological activity can be analyzed by relating the shape-dependent steric and electrostatic field of molecules to their biological activity. 

The results of a 3D-QSAR depend on a number of factors, each of which must be carefully considered. One of the most important considerations involves the selection of biologically active conformations and their alignment prior to the analysis. This may be relatively straightforward when one is working with a congeneric series of compounds that all have some key structural features that can be overlaid. For example, the original CoMFA paper [1] examined a series of steroid molecules which can be overlaid easily using the rigid steroid nucleus. In most cases, however, molecules of interest for drug discovery have flexible structural features and overlaying them objectively is not easy. 

 Expansion of the possibility of the 3D-QSAR is highly important to substantially promote many drug discovery projects where obtaining reliable X-ray structures of complexes between the active molecule and the relevant receptor is technically difficult. We have developed an automated 3D-QSAR method named AutoGPA in order to solve the above-mentioned difficult problem practically and make the 3D-QSAR easier to use by ordinary medicinal chemists.

A pharmacophore is defined as an ensemble of steric and electronic features that is necessary to ensure the optimal supramolecular interactions with a specific biological target and to trigger (or block) its biological response. Since biologically active molecules for the same active site should share the common interactions at the site, their active conformations should possess common three-dimensional arrangements of pharmacophores. It is hoped that the geometries of pharmacophore features common to many of the actives will contain information related to the important interactions between the bound conformations of the actives and the receptors. Therefore, it is naturally expected that the selection of the active conformations and overlaying them can be undertaken objectively by searching the arrangement of pharmacophore features that induce good overlay of the most active molecules. In AutoGPA, pharmacophores in a set of molecules with biological activities are fully exploited to find the conformations closely related to their biological activities and overlay them.

Applications of AutoGPA to three inhibitor-receptor systems have demonstrated that AutoGPA can automatically generate reliable 3D-QSAR models from the 2D chemical structures and the biological activities of sets of the inhibitors.

## 2. Method

**Figure 1:**
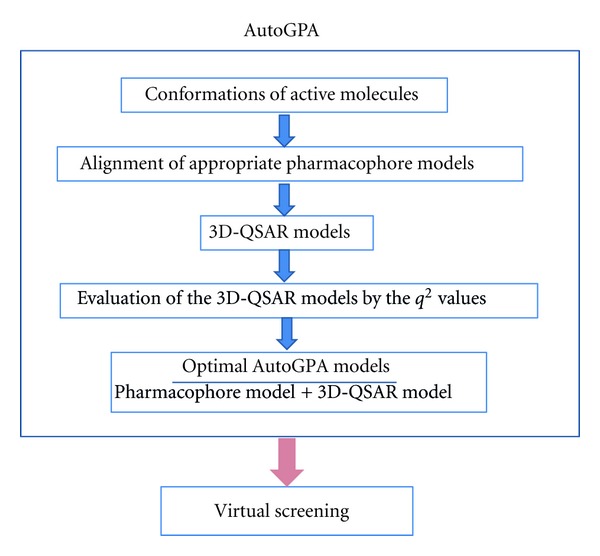
The process of developing AutoGPA models.

The software AutoGPA was coded by Scientific Vector Language implemented in Molecular Operating Environment (MOE) [2]. The process of AutoGPA is illustrated in Figure 1.

### 2.1. Pharmacophore-Based Alignments of Molecules

A function named conformation import implemented in MOE was applied to generate possible conformations with low strain energy for each molecule. The molecular mechanics setting using MMFF94x [3] force field with generalized Born solvation model [4] was applied. Typical pharmacophore features such as hydrogen bond acceptor, hydrogen bond donor, hydrophobic area, and positively and negatively ionized areas are assigned to each conformation. The three-dimensional arrangements of the pharmacophore features are compared for a set of biologically active molecules and the common 3D arrangements of pharmacophore features (hereafter pharmacophore queries) are extracted. The pharmacophore queries are used for selection of the conformations and their alignment prior to the 3D-QSAR modeling. The function named pharmacophore elucidation implemented in MOE can exhaustively search for all pharmacophore queries that induce good overlay of most of the active molecules and distinguish actives from inactives. This function is suitable for our purpose and is implemented in AutoGPA. Pharmacophore elucidation normally gives multiple possible alignments. Appropriate pharmacophores induce good alignment of the active molecules. Alignments are scored according to atomic overlap [5].

### 2.2. Building of 3D-QSAR Models

The algorithm of the comparative molecular field analysis (CoMFA) [1] is employed to develop 3D-QSAR models. Hydrogen atoms are added to all conformers of the molecules and the partial charges for all atoms are evaluated using the MMFF94x force field. A regular three-dimensional grid with a 2.0 Å separation surrounding all of the molecules is created. A “probe atom” is placed at each intersection grid point. An sp^3^ carbon atom with charge +1.0 is used as the probe atom. Molecular fields around each molecule are evaluated by calculating the electrostatic (Coulombic, with a 1/*r* dielectric) and steric (van der Waals 6–12) interaction energies between the molecule and a series of probe atoms placed at each grid point. The potential energy in kcal/mol is assigned to each grid point and a set of the grids is designated as a grid potential model in this paper. Wherever the probe atom experiences a steric repulsion greater than +30 kcal/mol, the relevant grid point is discarded from consideration. Other grid points named scoring grids are used to develop 3D-QSAR models. 

The partial-least-squares analysis is used to derive the 3D-QSAR models. The optimal number of components up to ten is identified by leave-one-out cross-validation. Each alignment yields the corresponding grid potential model and the cross-validated *r* ^2^(*q* ^2^). The set of pharmacophore model and the grid potential model that gives the best *q* ^2^ value is saved as an AutoGPA model for each alignment.

## 3. Results and Discussion

PDK1 is a promising target for developing anticancer drugs. Islam et al. have reported the inhibitory activities against PDK1 of 70 indolinone-based molecules [6, 7]. Using the chemical and biological data, Abdul-Hameed et al. have developed good CoMFA models [8]. They used the conformation of an inhibitor bound to PDK1 in the crystal structure (PDB ID: 2PE2) [7] of the complex between the inhibitor and PDK1 as a template for alignment of molecular structures. It means that they restricted the bioactive conformation from the beginning. In “real-world” situations, however, high-quality crystal structure of the complex between an active molecule and the target protein is not always available and identification of the bioactive conformation is usually extremely difficult. Practically this difficulty has severely limited the applicability of CoMFA and the objectivity of the results obtained. On the contrary, AutoGPA models can be obtained without prior knowledge of the three-dimensional molecular structures of the bioactive conformations. The greatest merit of AutoGPA lies in the fact that reasonably reliable 3D-QSAR models can be automatically obtained from a set of molecules with a suitable range of biological activities. It is interesting to compare the 3D-QSAR models obtained by CoMFA and AutoGPA.

**Figure 2:**
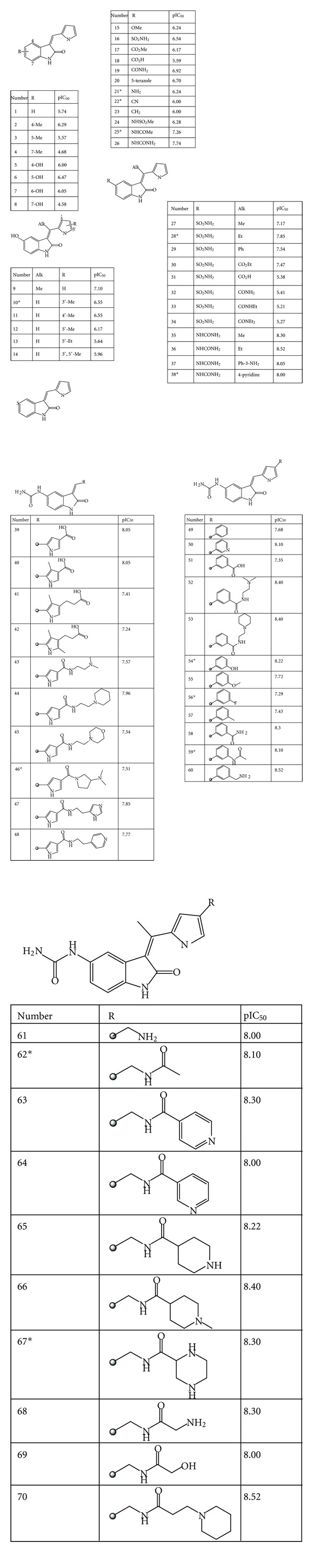
Chemical structures and the pIC50 values of PDK1 inhibitors used for validation of AutoGPA. The molecules used for a test set are asterisked, and other molecules are used for a training set.

**Figure 3:**
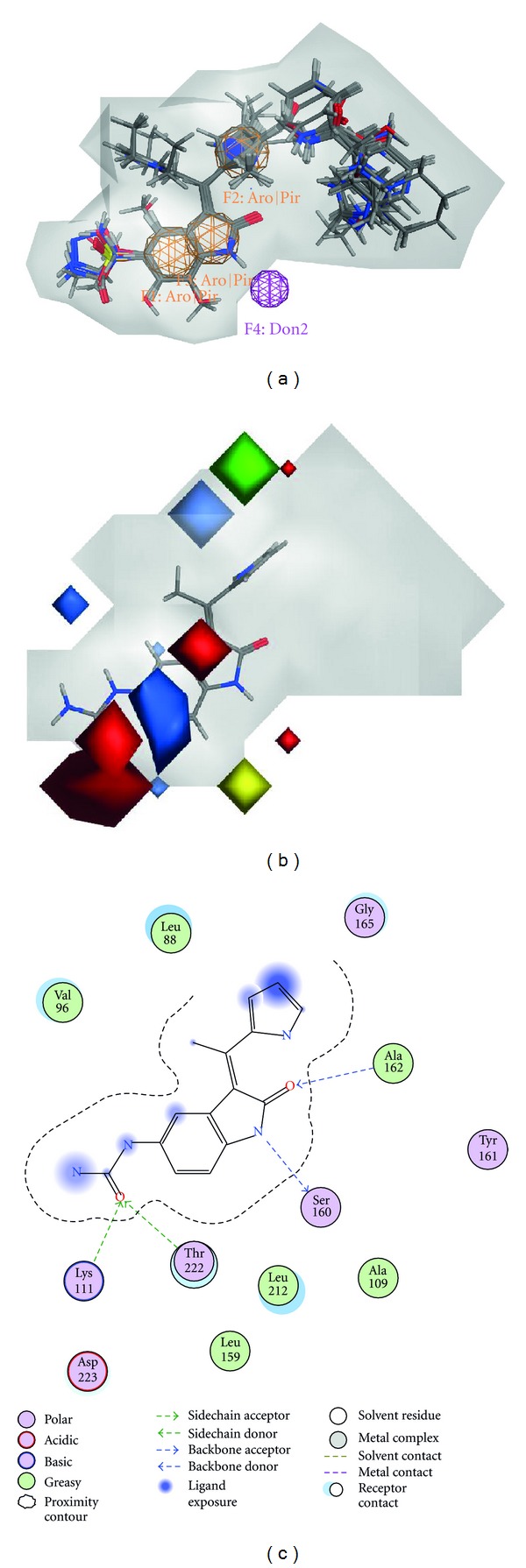
The best AutoGPA model obtained from the training set of PDK1 inhibitors. (a) The pharmacophore query of the best model is shown together with 56 molecules in the training set. (b) AutoGPA steric and electrostatic contours field plot. The position and the structure of molecule35 are superposed. Green and yellow contours indicate regions where bulky groups increase and decrease activity, respectively. Blue and red contours indicate regions where positive and negative electrostatic groups increase activity, respectively. (c) The interactions between the molecule 35 and PDK1 observed in the crystal structure (PDB code: 2PE1).

The dataset was separated into a training set for which AutoGPA models are derived and a test set that will prove the external predictivity of the resulting models. For comparison with the results by Abdul-Hameed et al. by CoMFA, we adopted the same training and test sets as they used. Representative molecules in the training and test sets are given in Figure 2 together with their pIC_50_ values. The molecules in the test set are asterisked. All molecules share a common chemical skeleton. Ten different AutoGPA models were generated and the results are summarized in Table 1. The statistics of the CoMFA model is also shown for comparison. In this table, various parameters that can assess the appropriateness of the models are given. The statistical parameters of the best model in terms of the cross-validated correlation coefficient (*q* ^2^) suggest that this model is reasonable and should have a good predictive ability. The *q* ^2^ and *r* ^2^ values are significantly higher and the mean squared error is significantly lower than those obtained for the CoMFA model. It is noteworthy that the AutoGPA model can also suggest the pharmacophores which are essential for ligand binding at the binding site of the expected target protein. The best AutoGPA model indicates that there are three aromatic or *π* ring centers, and a hydrogen donor. Such information about pharmacophore features at the binding site is helpful to grasp the chemical characteristics of bioactive molecules. In Figure 3(a), the alignment of the conformations of the molecules in the training set is shown together with the pharmacophore features. Three aromatic or *π* ring centers agree nicely to the corresponding three rings in the conformations. The satisfactory overlay clearly indicates that AutoGPA can successfully identify the bioactive conformation and the molecular alignment objectively in this case. In Figure 3(b), the molecular fields obtained by AutoGPA are depicted by steric and electrostatic contour maps. The conformation of molecule **35** is also shown in Figure 3(b). The combination of pharmacophore features and the grid potentials gives us an image of the characteristics of the binding site. In Figure 3(c), the molecular interactions observed in the crystal structure of the complex between molecule 35 and PDK1 [6] are illustrated. The pharmacophore features and the grid potentials generally correspond to the experimentally observed interactions. The projected hydrogen donor feature (Don2) shown in Figure 3(a) obviously corresponds to Ser160. The red polyhedra located over the urea group accept the approach of Lys111 and Thr222. The significant green polyhedron corresponds to the open space observed in the crystal structure of the complex. 

**Figure 4:**
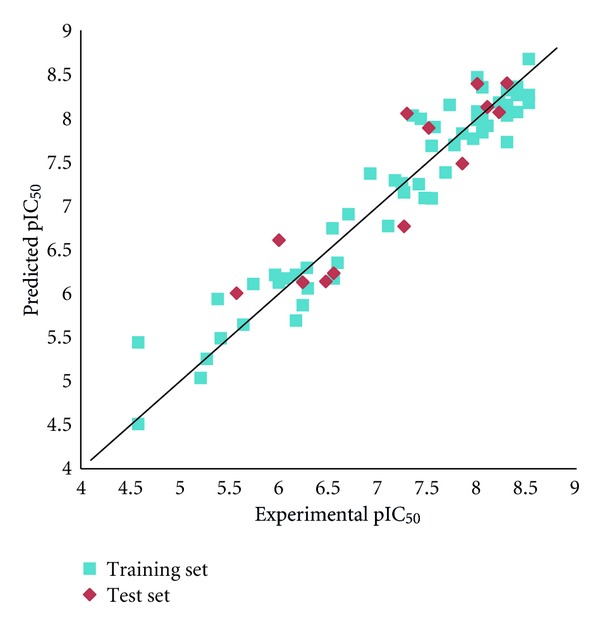
Plots of the experimental versus predicted pIC50 values using the training set of 56 molecules and the test set of 14 molecules.

The best AutoGPA model with a *q* ^2^ value of 0.760 was further validated by use of an external test set of 14 molecules. Since the biological activities of all the conformations which satisfy both the pharmacophore and grid potential models are evaluated in AutoGPA, multiple predicted values are assigned for each molecule. In this study, the maximum value is adopted as the predicted activity for each molecule. The correlations between observed and predicted pIC_50_ values are depicted in Figure 4. The best AutoGPA model predicts adequately the biological activities of molecules not included in the training set. The *r*_pred^2^_ value of 0.811 attained is almost identical to that of 0.812 obtained by the CoMFA model. Despite the fact that no prior knowledge about the three-dimensional structure of the active conformation is employed to develop the 3D-QSAR model, the predictive ability of AutoGPA is highly satisfactory.

 Additional two 3D-QSAR studies were undertaken to assess the performance of AutoGPA. The results will be concisely described below. The first study is on inhibitors of epidermal growth factor receptor (EGFR). Pasha et al. [9] developed 3D-QSAR model by CoMFA and CoMSIA. In this study, the X-ray structure of EGFR was used to obtain best-fit docking-based 3D-QSAR model. 46 and 12 molecules were used for training and test sets, respectively. The docked geometry-based CoMFA and CoMSIA models gave *q* ^2^ of 0.66 and 0.59, respectively, for the training set. For the test set, *r* ^2^ were 0.72 and 0.63, respectively. Although the AutoGPA model has given *q* ^2^ of 0.564 that is lower than those obtained by CoMFA and CoMSIA, *r* ^2^ of 0.768 is better than those obtained by CoMFA and COMSIA. Judging from the fact that AutoGPA used no three-dimensional information of the receptor in the analysis, the result indicates that AutoGPA gave a reasonably good 3D-QSAR model. The second study is on inhibitors of human dihydrofolate reductase (DHFR). Dixon et al. [10] introduced the software named PHASE and developed a 3D-QSAR model for a set of human DHFR inhibitors. Since PHASE is based on identification of common pharmacophore among active molecules and does not require experimentally determined three-dimensional structures of both ligands and receptors, it is conceptually similar to AutoGPA. They used training and test sets which comprise of 20 and 57 molecules, respectively. The *q* ^2^ value for the training set and the *r* ^2^ value for the test set are 0.932 and 0.492, respectively. The corresponding values obtained by AutoGPA are 0.910 and 0.500, respectively. The correlation coefficients obtained by PHASE and AutoGPA are only marginally different. However, the numbers of molecules ranked high are significantly different. Six molecules of the top 10 active molecules were predicted in the top 10 by AutoGPA, but only three active molecules were predicted in the top 10 by PHASE. In addition, the best molecule with the experimental pIC50 value of 8.57 was ranked 24 and 42 by AutoGPA and PHASE, respectively. These results obviously indicate that AutoGPA is relatively superior to PHASE in the prediction ability. 

In addition to PHASE, various ligand-based approaches employing pharmacophoric hypotheses have been reported in the literatures [11, 12]. Since in these approaches several different computer programs are used in combination, rather complicated tasks are required and relatively deep knowledge and experience on 3D QSAR are essential. On the contrary, the great merit of AutoGPA is that researchers, even novices on 3D QSAR, can obtain the comparable 3D QSAR only from a dataset of compounds with the chemical structures and the biological activities by executing a practically single job.

## 4. Conclusions

 The present study has clearly shown that AutoGPA can develop reliable 3D-QSAR models without prior knowledge about the three-dimensional structure of bioactive conformations. The results obtained for three inhibitor-receptor systems have demonstrated that AutoGPA can substantially resolve the problem of objective identification of bioactive conformations and their alignments that has severely limited the application of 3D-QSAR method such as CoMFA so far. Since prerequisite of AutoGPA is only a set of 2D structures of inhibitors with their biological activities, the analysis can be undertaken almost without human intervention. It is highly expected that AutoGPA can be practically applicable to various “real-world” drug discovery projects. 

## Figures and Tables

**Table 1:** Statistics of ten AutoGPA models based on the training set.

Model	Overlap	PH4	NOC	Grids	MSE	*r* ^2^	*q* ^2^
1	46.91	RRRd	6	28	0.092	0.923	0.760
2	46.45	RRHa	7	16	0.141	0.882	0.731
3	46.44	RRHd	4	28	0.214	0.821	0.724
4	46.83	RRda	4	22	0.174	0.854	0.695
5	46.91	RRRa	4	17	0.218	0.818	0.675
6	46.84	RRHd	3	21	0.252	0.789	0.661
7	46.27	RHda	4	25	0.201	0.832	0.660
8	46.49	RRda	3	19	0.233	0.805	0.650
9	46.92	RRRd	3	26	0.249	0.791	0.626
10	46.41	RHda	5	19	0.192	0.839	0.612

CoMFA∗	—	—	5	—	0.354	0.907	0.737

Overlap: atomic overlapping score in pharmacophore-based alignment.

PH4: pharmacophore feature labels; R: aromatic or *π*-ring center, H: hydrophobic, d: projected donor, a: projected acceptor.

NOC: number of components.

Grids: number of grid points for QSAR model.

MSE: mean squared error.

*r* ^2^: correlation coefficient.

*q* ^2^: predictive coefficient in leave-one-out cross-validation.

∗The CoMFA model obtained by Abdul-Hameed et al. [8].
